# Dextran-Sulfate Plasma Adsorption Lipoprotein Apheresis in Drug Resistant Primary Focal Segmental Glomerulosclerosis Patients: Results From a Prospective, Multicenter, Single-Arm Intervention Study

**DOI:** 10.3389/fped.2019.00454

**Published:** 2019-12-03

**Authors:** Rupesh Raina, Vinod Krishnappa, Cheryl Sanchez-Kazi, Alejandro Quiroga, Katherine E. Twombley, Robert Mathias, Megan Lo, Ronith Chakraborty, Shefali Mahesh, Julia Steinke, Timothy Bunchman, Joshua Zaritsky

**Affiliations:** ^1^Department of Nephrology, Cleveland Clinic Akron General and Akron Children's Hospital, Akron, OH, United States; ^2^Akron Nephrology Associates/Cleveland Clinic Akron General, Akron, OH, United States; ^3^Northeast Ohio Medical University, Rootstown, OH, United States; ^4^Department of Nephrology, Loma Linda University Children's Hospital, Loma Linda, CA, United States; ^5^Department of Nephrology, Spectrum Health (Helen De Vos Children's Hospital), Grand Rapids, MI, United States; ^6^Department of Pediatrics, Medical University of South Carolina, Charleston, SC, United States; ^7^Department of Pediatrics, Nemours Children's Hospital, Orlando, FL, United States; ^8^Department of Pediatrics, Children's Hospital of Richmond at VCU, Richmond, VA, United States; ^9^Department of Nephrology, Akron Children's Hospital, Akron, OH, United States; ^10^Division of Pediatric Nephrology, Dialysis and Transplantation, Helen Devos Children's Hospital and Clinics, Grand Rapids, MI, United States; ^11^Pediatric Nephrology and Transplantation, Children's Hospital of Richmond, Virginia Commonwealth University, Richmond, VA, United States; ^12^Nemours, A.I. duPont Hospital for Children, Wilmington, DE, United States

**Keywords:** focal segmental glomerulosclerosis, lipoprotein apheresis, nephrotic syndrome, liposorber, proteinuria

## Abstract

**Background:** Focal segmental glomerulosclerosis (FSGS) causes end stage renal disease (ESRD) in significant proportion of patients worldwide. Primary FSGS carries poor prognosis and management of FSGS patients, refractory to standard treatments or resistant to steroids, remains a major challenge. Lipoprotein apheresis is a therapeutic approach for drug resistant primary FSGS and post-renal transplant primary FSGS recurrence.

**Objectives:** To examine the safety and probable benefit at 1, 3, 6, 12, and 24-months following completion of apheresis treatment using Liposorber® LA-15 system in patients with nephrotic syndrome (NS), due to refractory primary FSGS or primary FSGS associated NS, in post renal transplant children.

**Material and Methods:** Prospective, multicenter, single-arm intervention study using Liposorber® LA-15 system. Patients ≤21 years old with drug resistant or drug intolerant NS secondary to primary FSGS with glomerular filtration rate (GFR) ≥60 ml/min/1.73 m^2^ or post renal transplant patients ≤21 years old with primary FSGS associated NS were included in the study. Each patient had 12 dextran-sulfate plasma adsorption lipoprotein apheresis sessions over a period of 9 weeks. All patients were followed up at 1, 3, 6, 12, and 24-months following completion of treatment.

**Results:** Of 17 patients enrolled, six were excluded from the outcome analysis (protocol deviations). Of the remaining 11 patients, all but one have completed apheresis treatments. Three patients were lost to follow-up immediately after completion of apheresis and excluded from outcome analysis. At one-month follow-up, 1 of 7 patients (14.3%) attained partial remission of NS while 2 of 4 subjects (50%) and 2 of 3 subjects (66.7%) had partial/complete remission at 3- and 6-months follow-up, respectively. One of two patients followed up for 12 months had complete remission and one patient had partial remission of NS after 24 months. Improved or stable eGFR was noted in all patients over the follow-up period.

**Conclusion:** The results of our multicenter study showed improvement in the response rates to steroid or immunosuppressive therapy and induced complete or partial remission of proteinuria in some of the patients with drug resistant primary FSGS. The main limitation of our study is the small number of subjects and high dropout rate.

## Background

Focal segmental glomerulosclerosis (FSGS) causes end stage renal disease (ESRD) in significant proportion of patients in the United States and worldwide (1, 2). FSGS is characterized by nephrotic or subnephrotic range proteinuria due to sclerosis and scarring of the glomerulus, which often progresses to ESRD (1, 3). Primary FSGS is idiopathic and commonly occurs in children and young adults, while secondary FSGS is frequently seen in older adults due to cytomegalovirus, human immunodeficiency virus (HIV) infection, systemic lupus erythematosus (SLE), sickle cell disease, hepatitis, reflux nephropathy, illicit drug use, and certain malignancies (1, 3). Pathology underlying FSGS is podocyte injury resulting in protein leak, capillary expansion, synechiae formation, and proliferation of mesangial matrix (3, 4). Proteinuria is the main presentation in FSGS and other symptoms are secondary to urinary protein loss, which include hypoalbuminemia, edema, hypertension, and hyperlipidemia (5, 6). Primary FSGS carries a poor prognosis as spontaneous remission is rare and it tends to rapidly progress to ESRD within 2–8 years in patients with persistent nephrotic range proteinuria (2, 7, 8).

The mainstay of treatment for primary FSGS is to control while preventing and decreasing the rate of progression to ESRD. Corticosteroids are most effective and commonly used treatment to induce remission of proteinuria in FSGS patients. However, remission rate is only 20–50% with steroid therapy for a mean duration of 3.7 ± 2 months (8, 9). Most steroid resistant FSGS patients develop ESRD (8). Furthermore, successful steroid therapy may come at the cost of severe adverse effects, such as growth impairment, hypertension, and immune suppression (6, 8). Steroid resistant or steroid intolerant FSGS patients can be treated with cyclophosphamide or a calcineurin inhibitor, but the response rates are very low (<25%) with significant complications (1, 8, 10). Moreover, long-term remission is uncertain in cyclophosphamide or calcineurin inhibitor responsive patients (1, 8).

FSGS recurs in 30–40% of renal transplant patients, and causes allograft injury in 20–30%, and graft loss in 40–50% of these patients (11). Furthermore, FSGS recurrence is the leading cause of graft failure in children and has the lowest 5-year graft survival rate for living donor renal transplant recipients compared to other renal disorders (7). Despite all the treatment options, management of FSGS patients, refractory to standard treatments or resistant to steroids, remains a major challenge. Lipoprotein apheresis therapy is used for drug resistant (corticosteroids and/or calcineurin inhibitors) primary FSGS and post-renal transplant primary FSGS reoccurrence. Lipoprotein apheresis selectively removes VLDL, LDL, and triglycerides without affecting serum HDL levels (12–14). Although underlying mechanism by which lipoprotein apheresis reduces proteinuria is unknown, several studies have shown that lipoprotein apheresis induces remission of drug resistant NS in FSGS patients (12, 14–19).

## Objectives

The primary objective was to examine the safety and potential benefit of lipoprotein apheresis at 1 month following a 9-week course of dextran-sulfate plasma adsorption lipoprotein apheresis for the treatment of patients with primary FSGS associated NS, who are refractory or intolerant to standard therapy, or primary FSGS associated NS, in post renal transplant children. The secondary objective was to examine the safety and potential benefit of dextran-sulfate plasma adsorption lipoprotein apheresis at 3, 6, 12, and 24-months following apheresis treatment in the same patient population.

## Materials and Methods

### Definitions Used in the Study

*Nephrotic syndrome* is defined as having a first morning void urine protein to creatinine ratio (UPCR) of >2 (g/g).

*Drug resistant NS due to FSGS* is defined as failure to attain partial or complete remission of NS with corticosteroids and/or calcineurin inhibitors (standard therapy) after at least 8 weeks of treatment in FSGS patients.

*Drug intolerant NS due to FSGS* is defined as patient not tolerating standard therapy due to severe adverse effects (such as growth impairment, hypertension, obesity, immune suppression, diabetes mellitus, etc.) with or without adequate clinical response.

*Partial remission of NS* is defined as first morning void sample UPCR of 0.2-2 (g/g) or decrease in UPCR ≥50% of initial screening value.

*Complete remission of NS* is defined as first morning void sample UPCR <0.2 (g/g).

### Study Design

Prospective, multicenter, single-arm intervention study of dextran-sulfate plasma adsorption lipoprotein apheresis using Liposorber® LA-15 system. Liposorber® LA-15 system has been described in the Appendix A (20). The study is registered on Clinical Trials.gov, which is a resource provided by the U.S. National Library of Medicine, and the number is as following: NCT02235857. The clinical sites (see Appendix A) have their Institutional Review Board (IRB) approval and they have been updated annually. Following FDA approval of the Liposorber® LA-15 system and the study plan in October 2013, the study was initiated in July 2014 with the enrollment completion in August 2018, expected follow up completion in October 2020, and expected final report submission in January 2021. Total duration of the study from initiation to completion of follow up will be 76 months. IRB approvals for the study from the respective clinical sites have been obtained. Informed consent from the patients or legal guardians of minor children (age <18 years) and assent from the children who can understand the language and procedure have been obtained. Sample size was calculated considering both primary safety and primary potential benefit objectives (*n* = 30). A maximum of 35 subjects from 3 to 10 sites will be enrolled in the study.

### Study Population

*Inclusion criteria:* Patients ≤ 21 years old with drug resistant or drug intolerant NS secondary to primary FSGS with glomerular filtration rate (GFR) ≥60 ml/min/1.73 m^2^ or post renal transplant patients ≤ 21 years old with primary FSGS associated NS. Patients with refractory or recurrent NS due to FSGS were also included if standard therapy was contraindicated. *Exclusion criteria:* Patients were excluded if they are >21 years old, pregnant, lactating or plan to conceive before completion of the study, unable to sign informed consent or adhere to follow up schedule, participating in another interventional study and weight <18 kgs. Patients were also excluded if they are on angiotensin converting enzyme (ACE) inhibitors that cannot be withheld for 24 h before each apheresis session, presently on other antihypertensives which cannot be withheld on the day of apheresis, life expectancy less than the study endpoint or medical condition that interferes with study schedule and outcome, known allergies to dextran sulfate or heparin or ethylene oxide, receiving vitamin K antagonists, severe hemophilia or hemorrhage diathesis, severe gastrointestinal ulcers, uncontrolled hypotension or hypertension, decompensated heart failure or valvular disease, unstable angina, acute myocardial infarction, uncontrolled cardiac arrhythmias, severe apoplexy, unresolved infection, hepatic or thyroid abnormalities.

### Study Protocol

Eligible patients were started on lipoprotein apheresis using Liposorber® LA-15 system. Each patient had a total of 12 apheresis sessions (twice/week for 3 weeks then once/week for 6 weeks) over a period of 9 weeks. Patients received medications at the discretion of the treating physician or as per institution's standard of care. Few exceptions were: ACE inhibitors should not be given for at least 24 h before each apheresis session (to avoid bradykinin release), other antihypertensives should be withheld on the day of each apheresis treatment until completion of the session. No initiation, discontinuation or routine changes in dosages of immunosuppressives (e.g., corticosteroids, calcineurin inhibitors, etc.), ACE inhibitors or angiotensin receptor blockers (ARBs) for 2 weeks before first apheresis treatment until 1 month following last apheresis treatment unless deemed absolutely medically necessary. Initiation of low dose corticosteroids (<0.3 mg/kg or <15 mg/day whichever is lower) can be considered for edema during the study period and was not interpreted as treatment failure. All the patients were followed up at 1, 3, 6, 12, and 24 months following the last apheresis session, and data was being collected as per Appendix A.

### Patient Participation Endpoints

All enrolled patients are expected to participate in the study for 24 months following last apheresis session. Participation will end if subject completes all the scheduled 24 month follow up visits, is lost to follow up, withdraws from the study, has reoccurrence of NS during 24 months follow up period, conceives during the study, requires plasmapheresis, develops ESRD, achieves complete remission at the time of baseline, death or study closure.

### Study Endpoints

The primary potential benefit endpoint was the percentage of subjects with partial or complete remission at 1-month post treatment, and the primary safety endpoint s the rate of device or procedure related serious adverse events (SAEs) happening during apheresis treatments and until 1 month after last apheresis session. Secondary endpoints were complete or partial remission or persistent NS at 1, 3, 6, 12, and 24 months following last apheresis session, and percentage of subjects who attained partial or complete remission at the 3, 6, 12, and 24 months follow up visits. Secondary endpoints also include incidence of adverse events (AEs) during apheresis treatments, incidence of all AEs and SAEs within 3, 6, 12, and 24 month follow up, and percentage of subjects showing an increase or decrease in lab values including percent change from baseline and at 1, 3, 6, 12, and 24 months follow up.

### Statistical Analysis

Clinically relevant baseline variables will be tabulated. Continuous variables will be reported as means and standard deviations and categorical variables will be reported as percents. Covariate analysis may be performed to identify predictors of SAEs and/or remission. Covariate analysis will also be performed with transplant status (i.e., pre-transplant, post-transplant) as a variable to identify additional predictors of SAEs and/or remission. Survival analysis techniques such as Kaplan-Meier or Cox Proportional Hazards will be incorporated if censoring of data occurs. Descriptive analysis of clinical parameters of NS (UPCR, serum total serum creatinine, eGFR, and LDL) stratified by the status of medication change (Yes vs. No) was provided.

## Results

**Figure 1:**
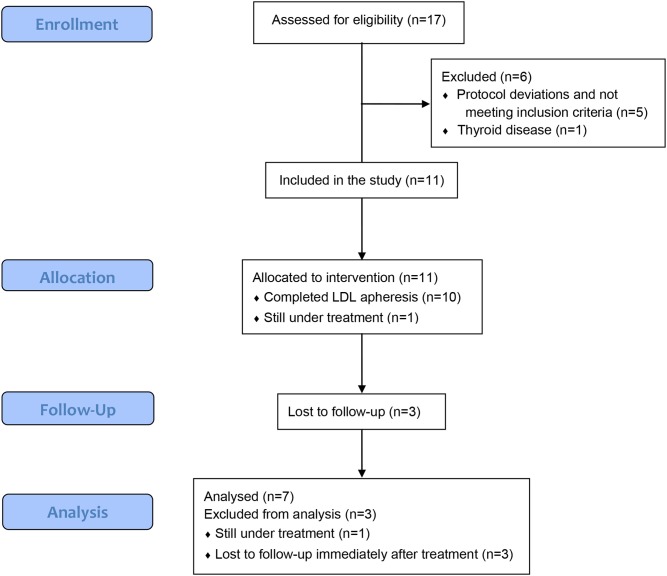
CONSORT flow diagram.

A total of 17 patients from 6 sites have been enrolled into the study. The majority of the patients are Caucasians (52.9%) with the maximum number in the age group 12–14 years (6/17, 35.3%) followed by 6–8 years (5/7, 29.4%) (Table 1). Of 17 patients, six were excluded from the outcome analysis; one patients who did not start treatment due to thyroid disease and five who received treatment but did not meet inclusion/exclusion criteria (protocol deviations due to UPCR <2) (Figure 1). Of remaining 11 patients, 10 had completed 12 apheresis treatments over a period of 9 weeks and one is still receiving apheresis treatments (Table 2). Of the 10 patients who completed apheresis treatments, three patients were lost to follow-up immediately after completion of apheresis and were excluded from outcome analysis (Table 2).

**Table 1:** Demographics of patients.

	***n* (%)**
**Age (years)**	
6–8	5 (29.4)
9–11	4 (23.5)
12–14	6 (35.3)
15–17	0
18–20	2 (11.8)
**Sex**	
Male	8 (47)
Female	9 (52.9)
**Race/ethnicity**	
Caucasian	9 (52.9)
African American	4 (23.5)
Hispanic/Latino	2 (11.8)
Unknown	2 (11.8)

**Table 2:** Follow-up visit data and nephrotic syndrome status for study subjects.

**Patient ID**		**Indication**	**Parameter**	**Baseline**	**After final treatment**	**1M F/U**	**3M F/U**	**6M F/U**	**12M F/U**	**24M F/U**	**Notes**
NCH001	Patient 1	Post-transplant	NS	NS	Partial	Partial	Partial	Partial	Withdrawal		Withdrawal after 6-month F/U
			UPCR (g/g)	44.33	13.02	17.43	12.81	17.51			
			SCR (mg/dl)	0.8	0.4	0.6	0.6	0.6			
			eGFR (ml/min/1.73 m^2^)	62.2	125.4	83.6	83.0	83.9			
			LDL (mg/dl)	60	71	269	344	498			
NDE001	Patient 2	Primary FSGS	NS	NS	NS	NA	NS	Withdrawal			Withdrawal after 3-month F/U
			UPCR (g/g)	8.11	3.84	Not performed	6.27				
			SCR (mg/dl)	0.7	0.7	0.7	0.8				
			eGFR (ml/min/1.73 m^2^)	89.4	91.0	89.7	78.7				
			LDL (mg/dl)	212	30	181	189				
NDE002	Patient 3	Primary FSGS	NS	NS	NS	NS	Partial	Complete	Complete	Par	24-month F/U completed
			UPCR (g/g)	6.33	<5.0	3.33	0.90	0.18	0.08	0.36	
			SCR (mg/dl)	0.8	0.4	0.6	0.6	0.7	0.7	0.7	
			eGFR (ml/min/1.73 m^2^)	84.9	172.2	112.9	114.3	98.3	100.3	100.4	
			LDL (mg/dl)	345	23	96	70	78	45	62	
NDE003	Patient 4	Post-Transplant Recurrence	NS		Not performed	Withdrawal					Withdrawal after final treatment (w/o F/U)
			UPCR (g/g)	5.05							
			eGFR (ml/min/1.73 m^2^)	95.8							
			LDL (mg/dl)	73							
NDE004	Patient 5	Primary FSGS	NS	NS	Excluded						Excluded from the study
			UPCR (g/g)	8.16							
			SCR (mg/dl)	0.7							
			eGFR (ml/min/1.73 m^2^)	72.9							
			LDL (mg/dl)	N/A							
NDE005	Patient 6	Post-Transplant Recurrence	NS	NS	NS	Withdrawal					Withdrawal after final treatment (w/o F/U)
			UPCR (g/g)	19.52	25.58						
			eGFR (ml/min/1.73 m^2^)	76.6	47.1						
			LDL (mg/dl)	64	47						
ACH001	Patient 7	Primary FSGS	NS	NS							Excluded from the study
			UPCR (g/g)	1.05	Excluded						
			SCR (mg/dl)	1.9							
			eGFR (ml/min/1.73 m^2^)	39.8							
			LDL (mg/dl)	165							
ACH002	Patient 8	Primary FSGS	NS	NS	NS	Partial	Complete	Partial	Complete	Complete	24-month F/U completed
			UPCR (g/g)	1.98	0.71	0.39	0.10	0.42	0.17	0.16	
			SCR (mg/dl)	0.3	0.3	0.4	0.4	0.4	0.4	0.5	
			eGFR (ml/min/1.73 m^2^)	170.7	170.3	129.1	129.8	130.1	132.2	109.4	
			LDL (mg/dl)	126	26	98	91	115	179	95	
ACH003	Patient 9	Primary FSGS	NS	NS	NS	NS	NS	NS	NS		Withdrawal after 12-month F/U
			UPCR (g/g)	1.81	3.48	2.67	2.11	4.01	3.78	Withdrawal	
			SCR (mg/dl)	1.2	1.2	1.4	1.2	1.4	2.2		
			eGFR (ml/min/1.73 m^2^)	60.0	60.0	51.9	60.9	52.5	33.9		
			LDL (mg/dl)	96	21	98	86	138	143		
ACH004	Patient 10	Primary FSGS	NS		Excluded						Excluded from the study
			UPCR (g/g)	0.08							
			SCR (mg/dl)	1.2							
			eGFR (ml/min/1.73 m^2^)	158.5							
			LDL (mg/dl)	103							
ACH006	Patient 11	Post-Transplant Recurrence	NS	NA							
			UPCR (g/g)	Not performed							Under treatment
			SCR (mg/dl)	0.7							
			eGFR (ml/min/1.73 m^2^)	72.7							
			LDL (mg/dl)	132							
LLU001	Patient 12	Post-Transplant Recurrence	NS	NS	NS	NS	Withdrawal				Withdrawal after 1-month F/U
			UPCR (g/g)	4.78	3.01	3.66					
			eGFR (ml/min/1.73 m^2^)	84.7	103.8	129.8					
			LDL (mg/dl)	N/A	4	81					
LLU002	Patient 13	Primary FSGS	NS	NS	NS	NS	Withdrawal				Withdrawal after 1-month F/U
			UPCR (g/g)	4.1	5.21	4.58					
			SCR (mg/dl)	0.3	0.3	0.3					
			eGFR (ml/min/1.73 m^2^)	153.0	159.1	160.8					
			LDL (mg/dl)	N/A	7	110					
LLU003	Patient 14	Post-Transplant Recurrence	NS	NS	NS	Partial	Partial	Partial	Partial	Partial	24-month F/U completed
			UPCR (g/g)	1.09	1.66	1.49	0.70	0.67	0.37	0.34	
			eGFR (ml/min/1.73 m^2^)	78.0	13	65.3	69.3	69.3	69.3	71.7	
			LDL (mg/dl)	44	13	100	78	86	115	103	
HDV001	Patient 15	Primary FSGS	NS	NA	NA						Withdrawal after final treatment (w/o F/U)
			UPCR (g/g)	Not performed	Not performed	Withdrawal					
			SCR (mg/dl)	0.7	0.6						
			eGFR (ml/min/1.73 m^2^)	83.2	98.8						
			LDL (mg/dl)	56	27						
CHS001	Patient 16	Primary FSGS	NS	NS	NA	NA	NS	NS	NS		12-month F/U completed
			UPCR (g/g)	5.42	Not performed	Not performed	12.51	8.83	2.77		
			SCR (mg/dl)	0.9	0.6	0.4	0.3	0.3	0.3		
			eGFR (ml/min/1.73 m^2^)	60.3	89.5	134.2	179.0	183.0	191.4		
			LDL (mg/dl)	N/A	15	389	370	350	163		
CHS002	Patient 17	Primary FSGS	NS	NS	NS	NS	Withdrawal				Withdrawal after 1-month F/U
			UPCR (g/g)	28.04	8.33	38.4						
			SCR (mg/dl)	0.3	0.4	0.5						
			eGFR (ml/min/1.73 m^2^)	216.1	163.5	130.8						
			LDL (mg/dl)	>DL^*4^	>DL^*4^	14						

*NS, nephrotic syndrome; F/U, follow up; UPCR, urine protein to creatinine ratio; SCR, serum creatinine; eGFR, estimated glomerular filtration rate; LDL, low density lipoprotein*.

One of the seven patients (14.3%) attained partial remission of NS at the 1-month follow-up visit and one patient whose UPCR data was missing during 1-month follow-up, had NS at the 3-month follow-up visit. Furthermore, 2 of 4 subjects (50%) and 2 of 3 subjects (66.7%) had partial/complete remission at 3- and 6-months following lipoprotein apheresis, respectively (Table 2). One out of two patients who were followed for 12 months had complete remission while one patient who was followed up for 24-months had partial remission of NS. Trends in eGFR and UPCR before (baseline) and after treatment are tabulated in Table 3. Improvement or stable eGFR was noted in all the patients (7/7, 100%) over the follow-up period. Details of steroids and immunosuppressive therapies are listed in Table 4. Reported side effects were nausea, vomiting, diarrhea, abdominal pain, fever/infection, pharyngitis, headache, lightheadedness, malaise, hypotension, leg cramps, allergic reaction, pneumonia, bacteremia, and anemia.

**Table 3:** Trends in eGFR and UPCR.

	**Baseline eGFR (ml/min/1.73 m^**2**^)**	**Last eGFR (ml/min/1.73 m^**2**^)**	**Δ eGFR (ml/min/1.73 m^**2**^)**	**Trend**	**Baseline UPCR**	**Last UPCR**	**Δ UPCR**	**Trend**
Patient 1	62	84	+22	Increase	44.3	17.5	−26.8	Decrease
Patient 2	89	79	−10	Stable	8.1	6.3	−1.8	Stable
Patient 3	85	100	+15	Increase	6.3	0.4	−5.9	Decrease
Patient 12	85	130	+45	Increase	4.8	3.7	−1.1	Stable
Patient 13	153	161	+8	Stable	4.1	4.6	+0.5	Stable
Patient 16	60	191	+131	Increase	5.4	2.8	−2.6	Decrease
Patient 17	28	38	+10	Increase	216	131	−85	Decrease

*eGFR, estimated glomerular filtration rate; UPCR, urine protein to creatinine ratio*.

**Table 4:** Experimental subject's medication list.

	**Medications at enrollment**	**During the course of LDL apheresis treatment**
Patient 1	Prednisone Mycophenolate mofetil Tacrolimus Amlodipine Losartan	No medication
Patient 2	Prednisone Tacrolimus Pravastatin Lisinopril	Losartan (Before 2nd session)
Patient 3	Prednisone Tacrolimus Losartan	No medication
Patient 4	Prednisone Losartan Lisinopril Mycophenolate mofetil	No medication
Patient 5	Data not available as treatment not started due to thyroid disease	N/A
Patient 6	Prednisone Valganciclovir Ketoconazole Bactrim Tacrolimus Amlodipine	Prednisone (Before 2nd session) Cyclophosphamide (Before 3rd session) Tacrolimus (Before 4th session) Amlodipine (Before 7th session) Mycophenolate mofetil (Before 9th session) Metolazone (Before 11th session)
Patient 7	Prednisone Pravastatin Tacrolimus Valsartan Amlodipine	No medication
Patient 8	Prednisone Cyclosporine Mycophenolate mofetil Lisinopril	Prednisone (Before 2nd and 12th session) Mycophenolate mofetil (Before 11th session)
Patient 9	Cyclosporine Simvastatin Enalapril Chlorothiazide Amlodipine	Losartan Amlodipine (Before 9th session)
Patient 10	Data not available as treatment was not performed	N/A
Patient 11	Prednisone Pravastatin Mycophenolate mofetil Cyclosporine Valacyclovir Clonidine Cozaar Amlodipine Bactrim Aranesp	Aranesp (Before 2nd session) Methylprednisolone (Before 8th session)
Patient 12	No medication	No medication
Patient 13	No medication	Tacrolimus (Before 6th session)
Patient 14	No medication	Mycophenolate mofetil (Before 10th session)
Patient 15	Prednisone Mycophenolate mofetil Tacrolimus Amlodipine Isradipine Labetalol	Tacrolimus (Before 3rd, 7th, 9th, 10th session)
Patient 16	Prednisone Mycophenolate mofetil Simvastatin Amlodipine Isradipine	Simvastatin (Before 12th session)
Patient 17	Prednisone Amlodipine	Cyclosporine (Before 5th, 7th, and 9th session) Prednisone (Before 8th, 9th, and 10th session) Losartan Amlodipine (Before 8th session) Mycophenolate mofetil (Before 12th session)

## Discussion

Our study showed that partial/complete remission rates of NS at 1, 3, 6, 12, and 24-month follow-up after completion of lipoprotein apheresis treatment were 14.3, 50, 66.7, 50, and 100%, respectively with stable or improvement in eGFR in all the patients. Management of steroid resistant primary FSGS is a challenge to date and bear poor prognosis given <25% response rate with uncertain long-term outcomes in patients treated with cyclophosphamide and calcineurin inhibitor therapy (1, 8, 10). Lipoprotein apheresis has been shown to improve the response rates to steroid or immunosuppressive therapy and induce complete or partial remission of proteinuria with histological recovery from the disease (12–19, 21–23). The mechanism by which lipoprotein apheresis produces favorable effects on the outcomes of primary FSGS is poorly understood, however, several theories have been put forth (24); (1) Improvement in macrophage function due to decrease in lipotoxic effect on glomeruli/interstitium as a result of reduction in lipid levels, (2) Better response to steroids and calcineurin inhibitor therapy as a result of lowering lipid levels, (3) improvement in endothelial dysfunction due to decrease in vascular cell adhesion molecule-1 (VCAM-1), (4) Better blood flow due to the removal of fibrinogen and other anticoagulants, (5) Vasodilation due to fall in thromboxane A2 levels and rise in the levels of vascular endothelial growth factor (VEGF), nitric oxide, bradykinin, and endothelial derived growth factor, (6) Anti-inflammatory effect due to reduction in LDL oxidation, C-reactive protein, intercellular adhesion molecule-1 (ICAM-1) and P-selectin, and (7) Reduced levels of vascular permeability factor in the circulation (24).

In the US, Liposorber LA-15® system received Humanitarian Device Exemption approval from FDA for lipoprotein apheresis in the treatment of primary FSGS in both adults and children who are refractory to standard treatment, have a GFR ≥60 mL/min/1.73 m^2^, or are post-renal transplantation (25). Our study showed reduced remission rate at 1-month follow-up (14.3 vs. 64%) compared to a study by Hattori et al. (12). However, the limitation of our study is the small number of subjects and high dropout rate. Hattori et al. studied effectiveness of lipoprotein apheresis monotherapy vs. combination of lipoprotein apheresis with prednisolone therapy in 11 children with NS secondary to primary FSGS unresponsive to cyclosporine (CsA) and steroids (12). Lipoprotein apheresis alone did not have significant impact on lowering LDL levels or reducing proteinuria, however, significant reductions were noted in total cholesterol and triglycerides levels. Combination therapy of lipoprotein apheresis with prednisolone induced partial/complete remission of NS in 7 of 11 (64%) children at 1 month following lipoprotein apheresis. Of these seven children, five achieved complete remission and had normal renal function at median of 4.4 years after lipoprotein apheresis, and remaining two had partial remission at 1 month after lipoprotein apheresis (stable renal function at 4.5 years follow up in one patient while the other one eventually developed ESRD after 7.8 years) (12).

The prospective observational survey on the long-term effects of lipoprotein apheresis on drug-resistant nephrotic syndrome (POLARIS) showed remission of NS (urine protein <1.0 g/day) in 21 of 44 (47.7%) patients followed for 2 years (26). Clinical parameters immediately after lipoprotein apheresis therapy that contributed significantly for favorable outcome were serum total protein (4.9 ± 0.7 g/dl), serum albumin (2.9 ± 0.8 g/dl), serum creatinine (1.2 ± 0.7 mg/dl), eGFR (61 ± 27.2 ml/min/m^2^), urine protein (1.7 ± 1.8 g/day), triglycerides (240.2 ± 156.3 mg/dl), total cholesterol (194.3 ± 65.6 mg/dl), LDL (83.1 ± 60.4 mg/dl), HDL (66.5 ± 18.3 mg/dl), fibrinogen (271.1 ± 77.2 mg/dl), and thrombin-antithrombin III complex (14.7 ± 38.6 ng/ml) (26). In addition, published literature has revealed few case studies showing remission of NS following dextran-sulfate plasma adsorption lipoprotein apheresis along with steroid and immunosuppressive therapies in children with drug resistant FSGS (14, 18, 19).

Available evidence from the published literature shows that lipoprotein apheresis enhances response rates to steroids and CsA in both adult and pediatrics NS patients as the effectiveness of lipoprotein apheresis monotherapy in the treatment of NS is not known (12, 16, 17, 21, 26). This is because VLDL and LDL decreases glucocorticoid receptor binding sites and impede glucocorticoid actions (24). Due to this, combination of lipoprotein apheresis with CsA or steroids or both is recommended by the Japanese Society of Pediatric Nephrology (27). In our study, most of the patients were on steroids and immunosuppressive therapies (Table 4). Additionally, maximum benefit can be achieved by starting lipoprotein apheresis early in the course of the disease process as response rates are better in patients with highly selective proteinuria and presence of advanced interstitial fibrosis indicates poor prognosis (12). Besides these benefits, a recent retrospective analysis involving five adult patients showed that lipoprotein apheresis prevented recurrence of FSGS in renal transplant recipients (28). Two sessions of lipoprotein apheresis was administered to all patients before renal transplantation except one patient who had single session (28). Survival of renal graft was noted in all patients without FSGS recurrence during 2–22 months of observation period following renal transplantation (28).

Common side effects that are associated with extracorporeal circulation can be encountered during lipoprotein apheresis, however, there are no serious adverse effects reported (27). ACE inhibitors should be discontinued during lipoprotein apheresis to avoid hypotensive events and shock (27). Restricting the use of lipoprotein apheresis therapy to children who weigh >30 kg is also recommended due to consumption of a significant amount of blood by the extracorporeal circuit (27). However, the lower weight limit for lipoprotein apheresis treatments for our study was recently set to 18 kg.

## Conclusion

FSGS is the main cause of ESRD among children with a high recurrence rate. Primary FSGS carries a poor prognosis as spontaneous remission is rare and it more rapidly progresses to ESRD. Despite all the available treatments, management of FSGS patients refractory to standard treatments remains a major challenge. Lipoprotein apheresis therapy may be beneficial for drug resistant (corticosteroids and/or calcineurin inhibitors) primary FSGS and post-renal transplant primary FSGS reoccurrence. Results of our multicenter study showed partial remission rate of NS was 14.3% at 1-month following lipoprotein apheresis. Furthermore, partial/complete remission rates at 3 and 6-month follow-ups were 50 and 66.7%, respectively. In addition, all patients showed stable or improved eGFR during the follow-up period. The main limitation of our study is small number of subjects and high dropout rate.

## Data Availability Statement

The raw data supporting the conclusions of this manuscript will be made available by the authors, without undue reservation, to any qualified researcher.

## Author Contributions

RR, VK, CS-K, AQ, KT, RM, ML, SM, and JZ contributed to the conception and design of the study. RR, VK, CS-K, AQ, KT, RM, ML, RC, SM, JS, TB, and JZ helped organize the database and wrote sections of the manuscript. All authors contributed to manuscript revision and read and approved the submitted version.

### Conflict of Interest

The authors declare that the research was conducted in the absence of any commercial or financial relationships that could be construed as a potential conflict of interest.

## Appendix

**Appendix A:** **IRB approval has been given by the following institutions**.

**Name of the Hospital**	**State**	**Principal Investigator**	**Address**
Akron Children's Hospital	OH	Dr. Rupesh Raina	130 W. Exchange Street Akron, OH 44302
Nemours/Alfred I. duPont Hospital	DE	Dr. Joshua Zaritsky	1600 Rockland Road, ARB 211 Wilmington, DE 19803
Loma Linda University Health	CA	Dr. Cheryl Sanchez-Kazi	11234 Anderson St. Room B-726 Loma linda, CA 92336
Medical University of South Carolina	SC	Dr. Katherine Twombley	135 Rutledge Avenue. Charleston, SC 29425
Helen DeVos Children's Hospital/ Spectrum Health	MI	Dr. Alejandro Quiroga	100 Michigan NE, Mail Code 038 Grand Rapids, MI 49503
Children's Hospital of Richmond at VCU	VA	Dr. Megan Lo	1000 E. Broad St., Richmond, VA 23219
